# Role of Chymase in the Development of Liver Cirrhosis and Its Complications: Experimental and Human Data

**DOI:** 10.1371/journal.pone.0162644

**Published:** 2016-09-16

**Authors:** Giovanni Sansoè, Manuela Aragno, Raffaella Mastrocola, Giulio Mengozzi, Erica Novo, Maurizio Parola

**Affiliations:** 1 Division of Gastroenterology, Humanitas Gradenigo Hospital, Torino, Italy; 2 Department of Clinical and Biological Sciences, University of Torino, Torino, Italy; 3 Clinical Biochemistry Laboratory, San Giovanni Battista Hospital, Torino, Italy; University of Navarra School of Medicine and Center for Applied Medical Research (CIMA), SPAIN

## Abstract

**Background:**

Tissue Angiotensin II (Ang-II), produced through local non ACE-dependent pathways, stimulates liver fibrogenesis, renal vasoconstriction and sodium retention.

**Aim:**

To highlight chymase-dependent pathway of Ang-II production in liver and kidney during cirrhosis development.

**Methods:**

Liver histology, portal pressure, liver and kidney function, and hormonal status were investigated in rat liver cirrhosis induced through 13 weeks of CCl_4_, with or without chymase inhibitor SF2809E, administered between 4^th^ and 13^th^ CCl_4_ weeks; liver and kidney chymase immunolocation and Ang-II content were assessed. Chymase immunohistochemistry was also assessed in normal and cirrhotic human liver, and chymase mRNA transcripts were measured in human HepG2 cells and activated hepatic stellate cells (HSC/MFs) *in vitro*.

**Results:**

Rats receiving both CCl_4_ and SF2809E showed liver fibrotic septa focally linking portal tracts but no cirrhosis, as compared to ascitic cirrhotic rats receiving CCl_4_. SF2809E reduced portal pressure, plasma bilirubin, tissue content of Ang-II, plasma renin activity, norepinephrine and vasopressin, and increased glomerular filtration rate, water clearance, urinary sodium excretion. Chymase tissue content was increased and detected in α-SMA-positive liver myofibroblasts and in kidney tubular cells of cirrhotic rats. In human cirrhosis, chymase was located in hepatocytes of regenerative nodules. Human HepG2 cells and HSC/MFs responded to TGF-β1 by up-regulating chymase mRNA transcription.

**Conclusions:**

Chymase, through synthesis of Ang-II and other mediators, plays a role in the derangement of liver and kidney function in chronic liver diseases. In human cirrhosis, chymase is well-represented and apt to become a future target of pharmacological treatment.

## Introduction

In chronic liver diseases, liver fibrosis progression depends on interactions among injured hepatocytes, inflammatory cells, and hepatic myofibroblast (MFs)-like cells that originate from activation of hepatic stellate cells (HSCs) or portal fibroblasts. These interactions imply angiotensin II (Ang-II) production by activated HSCs [1], Ang-II binding to angiotensin type 1 (AT1) receptors in myofibroblasts, and promotion of transcription of genes of extracellular matrix components, pro-fibrogenic cytokines, and collagenolysis inhibitors [2–4]. Therefore, angiotensin-converting enzyme (ACE) inhibitors or AT1 receptor antagonists attenuate experimental liver fibrosis [5, 6].

Endothelins promote liver fibrosis too. Three isoforms of endothelin bind to two receptors (ET_A_ and ET_B_) and both endothelins and its receptors are up-regulated in the fibrotic liver. Stimulation of ET_A_ receptors in HSCs promote an increase in intracellular-free calcium coupled with cell contraction, proliferation and stimulation of fibrogenesis [7].

In the kidney, Ang-II and endothelins have several effects. Ang-II constricts the efferent glomerular arteriole, resulting in preservation of glomerular filtration, but peritubular capillary hydrostatic pressure decreases and reabsorption of sodium and water in the tubular nephron increases. In addition, Ang-II causes sodium reabsorption in the proximal convoluted tubule through direct stimulation of AT1 receptors [8]. In patients with ascitic cirrhosis, renal plasma flow (RPF) and glomerular filtration rate (GFR) inversely correlate with plasma levels of endothelin-1 (ET-1) [9], and systemic infusion of ET-1 results in a prompt anti-natriuretic response [10].

Interstitial concentrations of Ang-II in normal heart and kidney are approximately 100-fold higher than in plasma [11], and most tissue Ang-II is synthesized locally and not taken up from the circulation [12]. Moreover, increased synthesis of ET-1 has been described at least in the cirrhotic liver [13].

Injection of ACE inhibitors into the renal artery shows that non-ACE-dependent pathways account for 70% of Ang-II production in this interstitial compartment [14]. Moreover, in myocardial extracts from humans and dogs, 90% of Ang-II-forming activity is accounted for by chymase (a serine endopeptidase) and not by ACE (a peptidyl-dipeptidase) [15], and chronic chymase inhibition attenuates the development of cardiac fibrosis and ventricular remodeling after experimental myocardial infarction [16].

Chymase, in heart, renal tubules and ubiquitous mast cells, converts angiotensin I (Ang-I) into Ang-II (so-called interstitial renin-angiotensin system [RAS]) [15], as well as ACE does it in the systemic RAS. In areas of chronic inflammation, chymase converts also big endothelin into ET-1 [17] and activates transforming growth factor beta (TGF-β) by stimulating its Ang-II dependent synthesis [18].

Since both Ang-II and ET-1 stimulate liver fibrogenesis, promote sodium retention and renal vasoconstriction, and are generated by chymase, we explore hepatic and renal content and localization of chymase in the experimental rat model of cirrhosis with ascites due to chronic carbon tetrachloride (CCl_4_) administration. Moreover, we investigate the role of chymase in progression of liver histological damage and hepatic and renal failure by taking advantage of chronic administration of SF2809E, a selective oral chymase inhibitor. Finally, chymase is searched for in tissue samples of normal and cirrhotic human liver and, *in vitro*, in human activated HSC/MFs and hepatoblastoma cells.

## Materials and Methods

Data collection in experimental ascitic liver cirrhosis (phase 1) is followed by immunohistochemical and biomolecular tests performed in normal and cirrhotic human liver and, *in vitro*, in cells derived from human cirrhotic liver (phase 2).

### Phase 1: *in vivo* experimental study

Thirty male adult Wistar rats with advanced liver damage and twenty male adult Wistar control rats were studied. Advanced liver damage was induced by CCl_4_ (Riedel de Haen, Sigma-Aldrich, Seelze, Germany) administered by gavage twice a week for 13 weeks [19]. Control rats were studied following a similar period of standardized diet. On the study day, all procedures were performed under general anesthetic (a mixture of intraperitoneal Ketavet 100 [Farmaceutici Gellini, Sabaudia, Italy] and Rompum [Xylazine, Bayer A.G., Leverkusen, Germany]). Rats were cared for in compliance with the European Council directives (No. 86/609/EEC) and with the Principles of Laboratory Animal Care (NIH No. 85–23, revised 1985). Animals were provided with currently accepted veterinary care daily; no animal died prior to the experimental endpoint despite the cirrhosis induction program; at the experimental endpoint rats were euthanized by exsanguination through the aorta (read later). This scientific project was approved by the Ethical Committee of the University of Torino (permit number: D.M. 94/2011-B). Meiji Seika Pharma Co., Ltd., Yokohama, Japan, provided SF2809E, an oral chymase inhibitor, after isolation from fermentation broth of the actinomycete strain SF2809, identified as Dactylosporangium sp. [20]. Six compounds having chymase inhibitory activity were isolated, with the highest inhibitory activity shown by compound VI, named SF2809E. It inhibits chymase at the IC_50_ of 0.014–0.081μM, whereas it does not inhibit cathepsin G and chymotrypsin at the concentration of 20μM (specific chymase inhibition) [20].

#### Animal groups

SF2809E was dissolved in Tween 80 to obtain two different solutions in the same volume of fluid (400 μl): F_10_ (10 mg/kg b.w.) or F_20_ (20 mg/kg b.w.). The animals were divided into five groups of ten rats: controls (group G1), controls receiving F_10_ three times a week for 9 weeks (G2), rats with ascitic cirrhosis caused by 13 weeks of CCl_4_ (G3), rats receiving CCl_4_ for 13 weeks but receiving also F_10_ or F_20_ three times a week all through the 5^th^ and into the 13^th^ week of CCl_4_ (nine-week of SF2809E) (G4 and G5). 4 rats (group G0) were sacrificed at the end of 4 weeks of CCl_4_ to assess the degree of liver histological damage that preceded SF2809E administration.

#### Study protocol

G1-G5 rats, after respective weeks of treatment or observation, were anesthetized [19], laparotomy was performed, and the urinary bladder was emptied before clamping the urethral orifice for further urine collection. Shortly thereafter, infusion of inulin (IN) 10% (W/v) (Laevosan-Gesellschaft, Linz/Donau, Austria) and para-aminohippurate (PAH) 20% (W/v) (Nephrotest, BAG Gmbh, Munich, Germany) into the caudal vein was started to assess glomerular filtration rate (GFR) and renal plasma flow (RPF) by steady-state plasma clearances of IN and PAH (CIN and CPAH, respectively) [19, 21]. After the start of IN and PAH infusion, a polypropylene catheter (0.7 mm diameter) was inserted into a small ileal vein, gently advanced to the bifurcation of the superior mesenteric and the splenic veins, and portal pressure was measured [22]. When IN and PAH steady-state plasma concentrations had been reached [19], cardiac blood was sampled to assess plasma osmolality and concentrations of IN, PAH, sodium, potassium, bilirubin, albumin, aspartate aminotransferase (AST), alanine aminotransferase (ALT), vasopressin (ADH), plasma renin activity (PRA), Ang-II, and norepinephrine (N). Finally, urinary bladder was emptied to measure 90-min urine volume, osmolality and sodium and potassium excretion rates. Rats were then euthanized by exsanguination through the aorta. Five anesthetized rats in each group had their mean arterial pressure evaluated through tail sphygmomanometry [22] prior to any surgical procedure. After sacrifice, liver and kidneys of all rats were removed for further biomolecular studies. Rats belonging to G0 had their livers just removed for assessment of matrix deposition through α smooth muscle actin (αSMA) indirect immunofluorescence and Sirius Red staining.

#### Liver Sirius Red staining

Sirius Red staining was performed on formalin-fixed paraffin-embedded liver sections (2 μm thick) with rapid exposure to Harry’s hematoxylin to stain nuclei after staining in 0.1% Sirius Red F3B (Sigma—Aldrich, St. Louis, MO, USA). Computer based morphometric quantification of liver fibrosis in groups G1-G5 was then performed [23].

#### Liver αSMA immunohistochemistry

Immunohistochemistry was performed on paraffin liver sections (6 μm thick) with mouse monoclonal anti-αSMA (Sigma-Aldrich, Milan, Italy) [24].

#### Liver Gomori trichrome staining

Gomori trichrome staining, with Engel-Cunningham modifications, was performed [25].

#### Chymase protein concentrations in liver and kidney

For western blot analysis, blots were incubated with goat polyclonal chymase antibodies (Santa Cruz Biotechnology, Inc.) and antibodies against β-actin (Sigma, St. Louis, MO, USA) [24, 26]. The intensity of chymase bands in each experiment was normalized to the intensity of the corresponding β-actin band, used as internal standard of non-specific protein content.

#### Chymase indirect immunofluorescence in liver and kidney

Goat polyclonal anti-chymase (Santa Cruz Biotechnology, Inc.) or mouse monoclonal anti-αSMA (Sigma-Aldrich, Milan, Italy) primary antibodies were used [26]. Secondary antibodies were anti-goat C3y-conjugated antibodies (Amersham Biosciences, Braunschweig, Germany). In the kidney, anti-αSMA antibodies were employed to highlight chymase in relation to the wall of renal arterioles. DNA fluorescent dye DAPI was used to stain nuclei [27].

#### Liver and kidney chymase immunohistochemistry

Sections were incubated with rabbit polyclonal antibodies against chymase (Bioss Inc., Woburn, MA, USA) and standard procedures were applied [24].

#### Endothelin-1, Ang-II and TGF-β tissue concentrations (liver and kidney)

ET-1 and Ang-II levels in tissue homogenates were determined by RIA (Phoenix Pharmaceuticals, Inc., Karlsruhe, Germany) and through standard HPLC methods, respectively. Tissue levels of TGF-β were determined through ELISA, (TGF-β Abcam Kit—code n° ab119558; Cambridge, MA, USA).

#### Plasma and urine analyses

Plasma and urine concentrations of electrolytes, and IN and PAH plasma concentrations were measured [19]. Plasma ADH, N and PRA were determined as described elsewhere [28]. Plasma Ang-II levels were determined by enzyme immunoassay (EIA) (RayBiotech Inc., Norcross, GA, U.S.A.). Plasma transaminases, albumin and total bilirubin levels were determined with automated Roche/Hitachi Cobas equipment.

#### Calculations

Sodium and potassium clearances (CNa and CK), filtration fraction (FF), and fractional excretion of sodium (FENa) and potassium (FEK) were calculated [28]. CIN and CPAH were calculated through the steady-state plasma clearance formula as:

Cx=infusion rate(x)/ssP-x

where ssP-x is the steady-state plasma concentration of x. CIN and CPAH were taken as measures of GFR and RPF, respectively [21]. Tubular free-water reabsorption (TF-WR) was also calculated [29], through the formula:

TF−WR=Cosm−V

where V is the urinary output (ml/min); Cosm is the osmolar clearance, which was computed via the formula:

Cosm=(UosmxV)/Posm

where Uosm and Posm are urine and plasma osmolalities. Mean arterial pressure (MAP) was calculated from the formula [22]:

1/3 (systolic blood pressure–diastolic blood pressure) + diastolic blood pressure.

### Phase 2: data collection in human liver cirrhosis

#### Human liver chymase Immunohistochemistry

Immunohistochemistry was performed on paraffin-embedded sections from: a) livers explanted prior to liver transplantation from patients with hepatitis C virus (HCV)-related cirrhosis; b) surgical liver sections from patients without cirrhosis but with hepatic metastases from colorectal carcinoma. Immunohistochemistry in these sections of "controls" was performed in areas placed 3 cm off the edge of metastases. Human liver specimens were obtained after written informed consent from each patient. The use of human material conforms to the ethical guidelines of the 1975 Declaration of Helsinki and was approved for this study by the University of Torino Bioethical Committee. Sections were incubated with polyclonal anti-chymase antibodies (Bioss BS-2353R). After blocking endogenous peroxidase activity with 3% hydrogen peroxide and performing microwave antigen retrieval, primary antibodies were labeled by using EnVision, HRP-labeled System (DAKO) and visualized by 3’-diaminobenzidine substrate. For negative controls the primary antibodies were replaced by isotype- and concentrations-matched irrelevant antibody.

#### Quantitative real-time RT-PCR

Chymase mRNA levels were measured *in vitro* by real-time PCR in human HepG2 and HSC/MFs cells treated with TGF-β1 (10 ng/ml) up to 24 hours, using SoFast^™^ EvaGreen^®^ Supermix (Biorad) following manufacturer’s instructions. Real-time PCR was performed using MiniOpticon Real Time PCR System. Oligonucleotide sequence of primers used for RT-PCR were: sense 5’-GGGACTATCCACCTGCAAGA-3’; reverse 5’- CCTCCTTGGCGTAGTAGTCG -3’. The relative mRNA expression level was calculated by the threshold cycle (Ct) value of each PCR product and normalized with that of Beta-Actin by using comparative 2-ΔΔCt method.

#### Statistical analysis

All comparisons were made by one-tailed Wilcoxon rank sum test for paired or unpaired data, as needed. Results are expressed as means ± SD. Significance is accepted at the 5% probability level.

## Results

### Influence of chymase on liver fibrogenesis and development of cirrhosis

**Fig 1:**
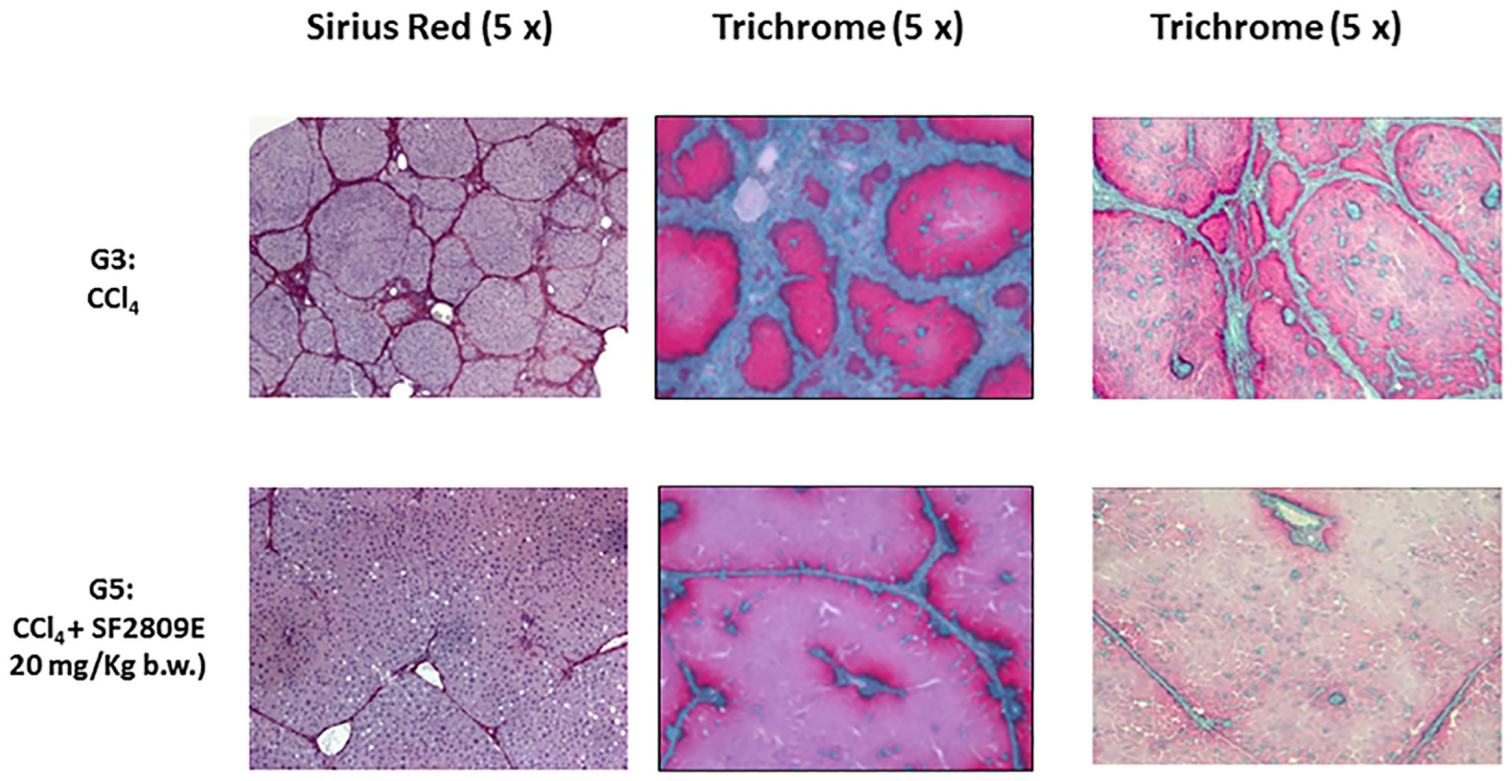
Morphological analysis of chronic liver disease progression. 13 weeks of CCl_4_ (G3): development of liver cirrhosis (and ascites). The liver of rats receiving both CCl_4_ and the chymase inhibitor was characterized by a significant prevention of fibrosis progression towards cirrhosis. This was maximal in animals treated with 20 mg/kg b.w. of the chymase inhibitor (G5). Prevention of fibrosis progression is clearly documented by either Gomori trichrome staining or Sirius Red.

**Fig 2:**
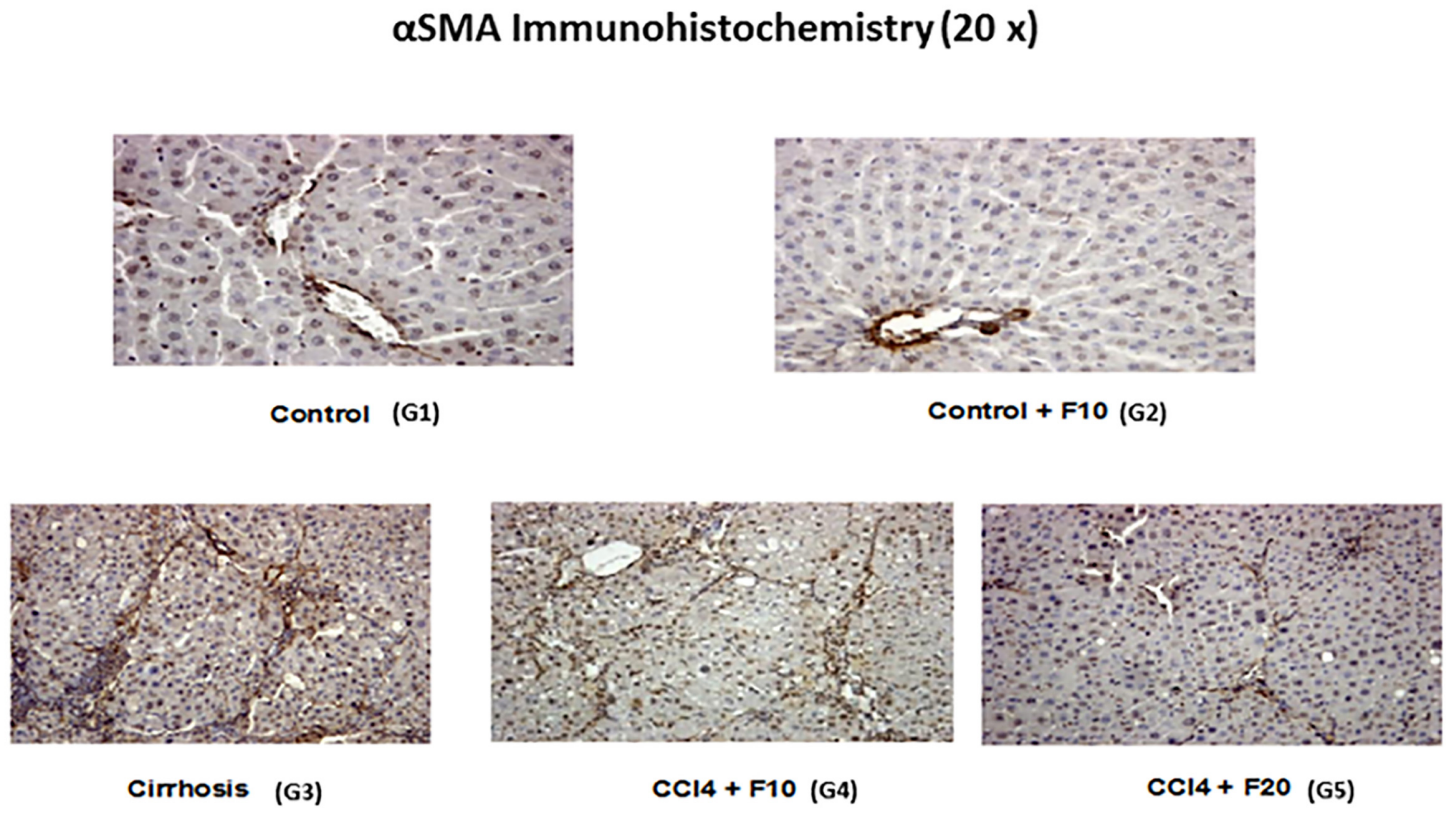
Morphological analysis of chronic liver disease progression through αSMA immunohistochemistry. Administration of chymase inhibitor to healthy rats (G2) did not affect normal liver morphology. 13 weeks of CCl_4_ (G3): development of liver cirrhosis (and ascites). The liver of rats receiving both CCl_4_ and the chymase inhibitor was characterized by a significant prevention of fibrosis progression towards cirrhosis. This, already appreciable in G4, was maximal in animals treated with 20 mg/kg b.w. of the chymase inhibitor (G5).

**Fig 3:**
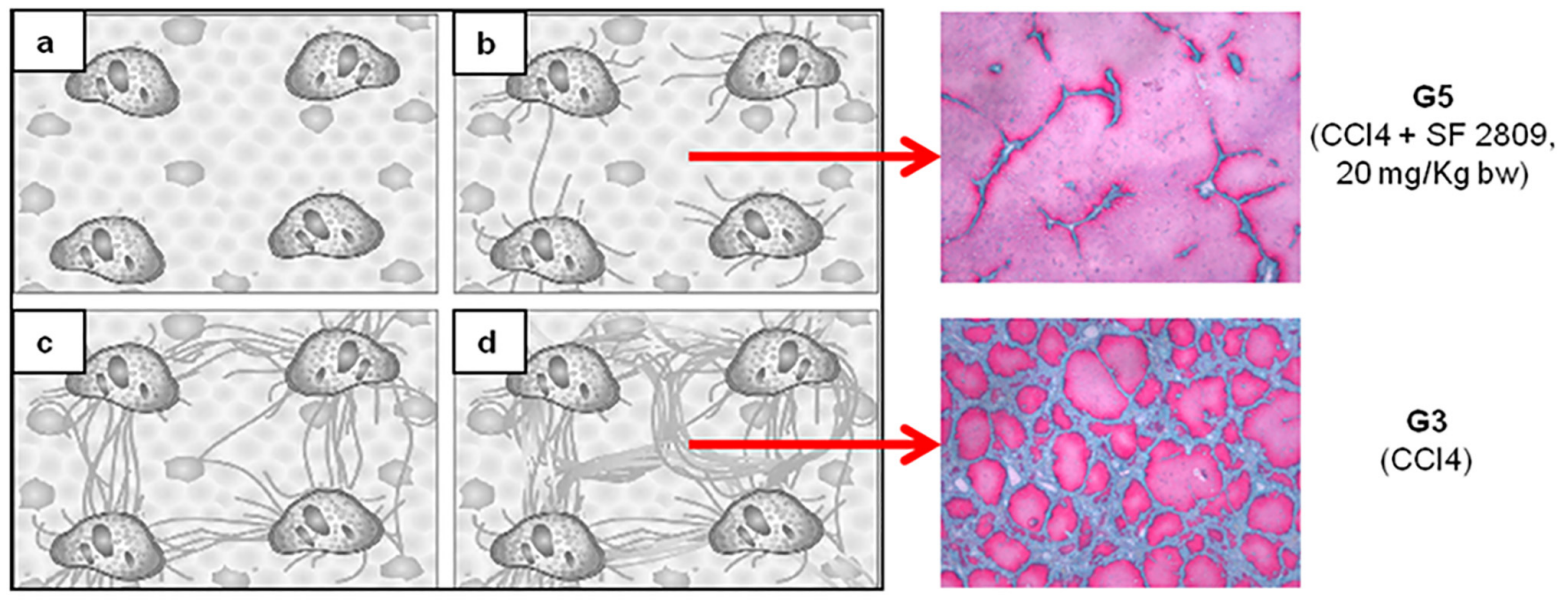
Batts-Ludwig diagram of progression of scarring in chronic liver diseases (reprinted with permission from Am J Surg Pathol 1995; 19: 1409–1417). The beneficial effects of chymase inhibition limit fibrosis progression to stage B scarring in G5, as compared to the stage D scarring (i.e. overt cirrhosis) detected in rats receiving CCl_4_ only (G3).

**Fig 4:**
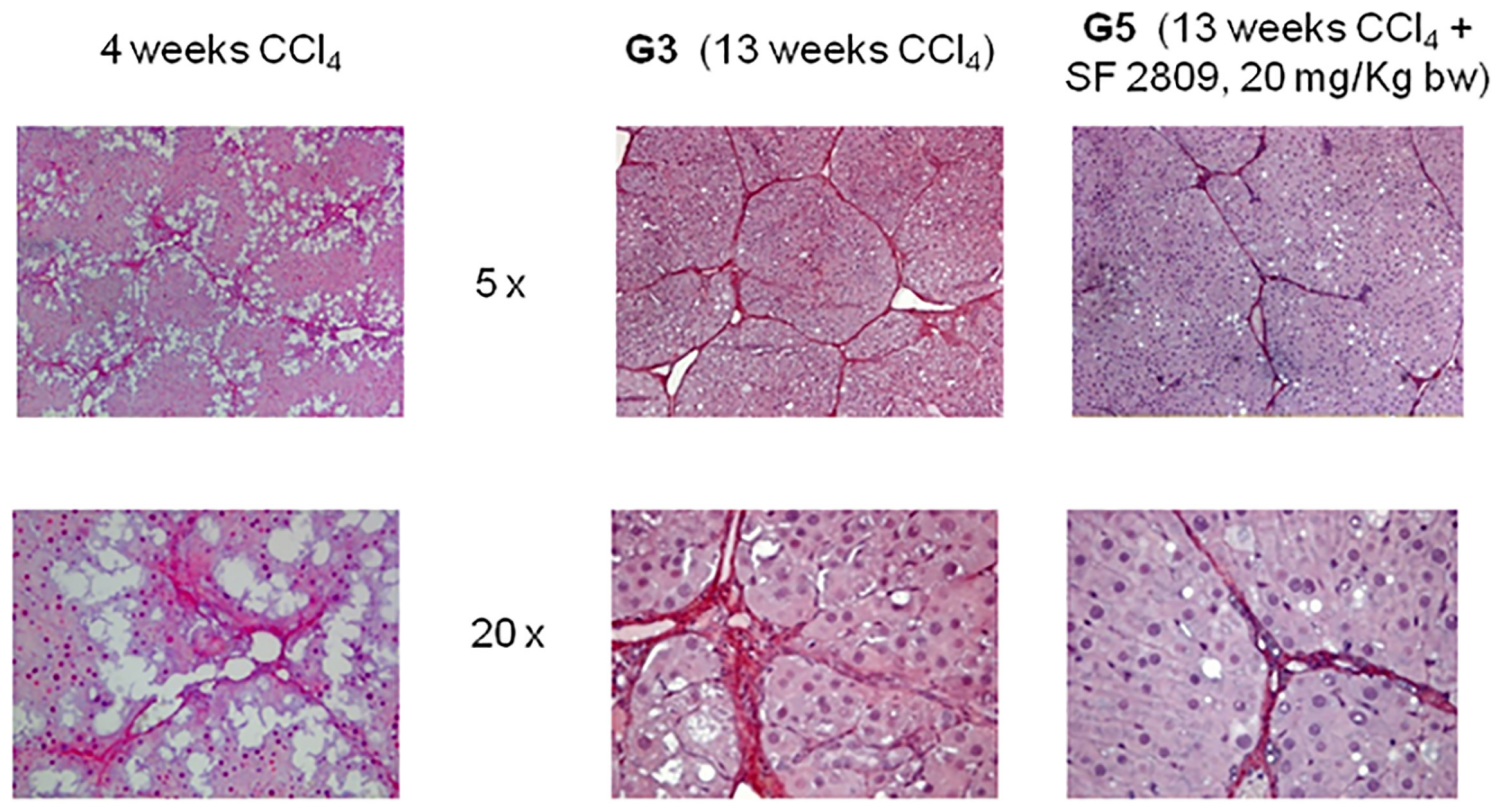
Once the treatment with chymase inhibitor at higher dosage was started (after 4 weeks of CCl_4_), Sirius Red staining confirms just a modest progression of liver fibrosis occurring in G5 group vs G3 group.

13 weeks of CCl_4_ resulted in cirrhotic ascites in all animals belonging to group G3; rats treated with both CCl_4_ and 20 mg/kg b.w. of the chymase inhibitor (G5) were devoid of ascites and had smooth livers of normal weight (Table 1). In group G3 kidneys were hypertrophic but of normal weight in G5 (Table 2). Gomori trichrome, Sirius Red (Fig 1) and αSMA immunohistochemistry (Fig 2) showed liver cirrhosis with thick fibrotic septa in G3 rats, micronodular cirrhosis in rats receiving 10 mg/kg b.w. of the chymase inhibitor (G4), fibrosis with architectural distortion in rats receiving both CCl_4_ and the larger dose of the chymase inhibitor (G5) [30]. Batts-Ludwig classification of liver scarring [31] confirmed stage B scarring in G5, in comparison with the stage D scarring (liver cirrhosis) displayed by G3 rats (Fig 3). Comparing liver histological damage caused by 4 weeks of CCl_4_ in G0 vs. G5 rats, limited progression of liver fibrosis occurred when SF2809E was used in high dosage (Fig 4). Morphometric computer-based assessment of fibrosis showed 0.13 ± 0.037% fibrosis-index in the control group (G1). A strong accumulation of collagen fibers was observed in rats treated with just CCl_4_ (G3) (4.75 ± 0.35%, P<0.01 vs. G1). This was much less evident in G5 rats (2.1 ± 0.28% fibrosis index, P< 0.03 vs. G3) [27].

**Table 1:** Liver weight and function data, portal pressure, and hepatic tissue levels of Ang II and ET-1 in the different rat groups.

	G1 (n = 10)	G2 (n = 10)	G3 (n = 10)	G4 (n = 10)	G5 (n = 10)
Liver weight (g)	10.1 ± 1.8	9.8 ± 1.5	8.9 ± 1.1^d^	9.4 ± 1.5	10.1 ± 1.6
Liver-to-body weight ratio (%)	2.4 ± 0.8	2.3 ± 0.9	2.6 ± 1.3	3.0 ± 1.9	3.0 ± 2.0
TGF-β (ng/mg liver prot.)	10.8 ± 2.1	11.2 ± 3.1	19.9 ± 2.7^a^	21.3 ± 4.1^a^	14.2 ± 3.2
Bilirubin (mg/dl)	0.2 ± 0.04	0.2 ± 0.03	2.8 ± 0.3^a^	1.7 ± 0.3	1.0 ± 0.2
Albumin (g/dl)	3.5 ± 1.1	3.7 ± 1.2	2.2 ± 0.9^a^	2.9 ± 0.9	3.0 ± 0.9
AST (UI/L)	51 ± 10	56 ± 16	143 ± 56^b^	106 ± 50^b^	174 ± 39^b^
ALT (UI/L	24 ± 7	22 ± 2	69 ± 27^b^	47 ± 22	66 ± 39^b^
Portal pressure (mmHg)	6.2 ± 1.1	5.3 ± 0.8	24.3±2.05^a^	19.0 ± 1	10.0±0.98
Ang II (pg/mg of total liver protein)	48 ± 10	52 ± 31	1400 ± 149^b^	1209 ± 122	260 ± 99^y^
ET-1 (pg/mg of total liver protein)	19.9 ± 3.7	16.6 ± 5.3	82.9 ± 16^a^	63.5 ± 17	63.7 ± 12

Data are means ± SD. G1: control rats; G2: controls receiving 10 mg/kg b.w. SF2809E; G3: cirrhotic rats; G4: rats receiving both CCl_4_ and 10 mg/kg b.w. SF2809E; G5: rats receiving both CCl_4_ and 20 mg/kg b.w. SF2809E.

^a^P<0.01 versus G1 and G5.

^b^P<0.01 versus G1.

^d^P<0.03 versus every other group.

^y^P<0.03 versus G3. (Wilcoxon rank sum test).

TGF-β, transforming growth factor beta; AST, aspartate aminotransferase; ALT, alanine aminotransferase; Ang-II, Angiotensin II; ET-1, endothelin-1.

**Table 2:** Kidney weight, arterial pressure, renal function, and systemic and renal hormonal levels in the different rat groups.

	G1 (*n* = 10)	G2 (*n* = 10)	G3 (*n* = 10)	G4 (*n* = 10)	G5 (*n* = 10)
Kidney weight (g)	1.8 ± 0.08	1.8 ± 0.09	2.3 ± 0.09^e^	1.8 ± 0.06	2.0 ± 0.09
Kidneys-to-body weight ratio (%)	0.8 ± 0.1	0.8 ± 0.2	1.3 ± 0.2 ^e^	1.0 ± 0.2	1.1 ± 0.4
MAP (mm Hg)	87 ± 9	88 ± 10	79 ± 15	83 ± 12	85 ± 12
TGF-β (ng/mg kidney prot.)	23.7 ± 5.4	20.1 ± 3.3	35.2 ± 5.3^e^	25.1 ± 4.1	22.1 ± 4
Ang II (pg/mg of total liver protein)	70 ± 21	78 ± 22	1150 ± 199^c^	999 ± 187	570 ± 91^a^
ET-1 (pg/mg of total liver protein)	2.02 ± 0.2	1.86 ± 0.1	1.99 ± 0.2	1.63 ± 0.2	1.45 ± 0.2^a^
Diuresis (ml/h)	1.0 ± 0.1	0.9 ± 0.1	0.8 ± 0.1^d^	0.6 ± 0.07	1.1 ± 0.1^a^
Natriuresis (μmol/h)	92 ± 7	89 ± 9	81 ± 6^d^	86 ± 7	105 ± 15^a^
FENa (%)	2.3 ± 0.2	2.1 ± 0.2	1.9 ± 0.1^d^	2.0 ± 0.2	3.1 ± 0.4^a^
Kaliuresis (μmol/h)	82 ± 16	78 ± 18	64 ± 34	47 ± 10	141 ± 15^a^
TF-WR (ml/h)	1.14 ± 0.3	1.12 ± 0.4	2.77 ± 0.7^c^	2.55 ± 0.8	1.67 ± 0.4^a^
GFR (ml/min/g of kidney tissue)	0.16 ± 0.03	0.16 ± 0.05	0.11 ± 0.02^c^	0.12 ± 0.02	0.13 ± 0.02^b^
FF (%)	45 ± 4	43 ± 4	41 ± 2^d^	38 ± 3	64 ± 5^a^
PRA (ng/ml/h)	20.4 ± 2.2	19.3 ± 2.0	22.2 ± 1.9^c^	21.7 ± 1.7	20.6 ± 1.1^a^
Plasma Ang-II (pg/ml)	246 ± 51	164 ± 68	358 ± 61^c^	320 ± 228	271 ± 42^a^
Plasma ADH (pg/ml)	240 ± 34	221 ± 39	359 ± 41^c^	350 ± 44	275 ± 39^a^
Plasma N (ng/ml)	202 ± 68	231 ± 71	249 ± 59^c^	252 ± 51	166 ± 51^a^

Data are means ± SD. G1: control rats; G2: controls receiving 10 mg/kg b.w. SF2809E; G3: cirrhotic rats; G4: rats receiving both CCl_4_ and 10 mg/kg b.w. SF2809E; G5: rats receiving both CCl_4_ and 20 mg/kg b.w. SF2809E.

^a^P<0.01 versus G3.

^b^P<0.05 versus G3.

^c^P<0.01 versus G1.

^d^P<0.05 versus G1.

^e^P<0.03 versus every other group. (Wilcoxon rank sum test).

MAP, mean arterial pressure; TGF-β, transforming growth factor beta; Ang-II, Angiotensin II; ET-1, endothelin-1; FENa; fractional excretion of sodium; TF-WR, tubular free-water reabsorption; GFR, glomerular filtration rate; FF, filtration fraction; PRA, plasma renin activity; N, norepinephrine; ADH, vasopressin.

### Liver and kidney chymase content and immunostaining

**Fig 5:**
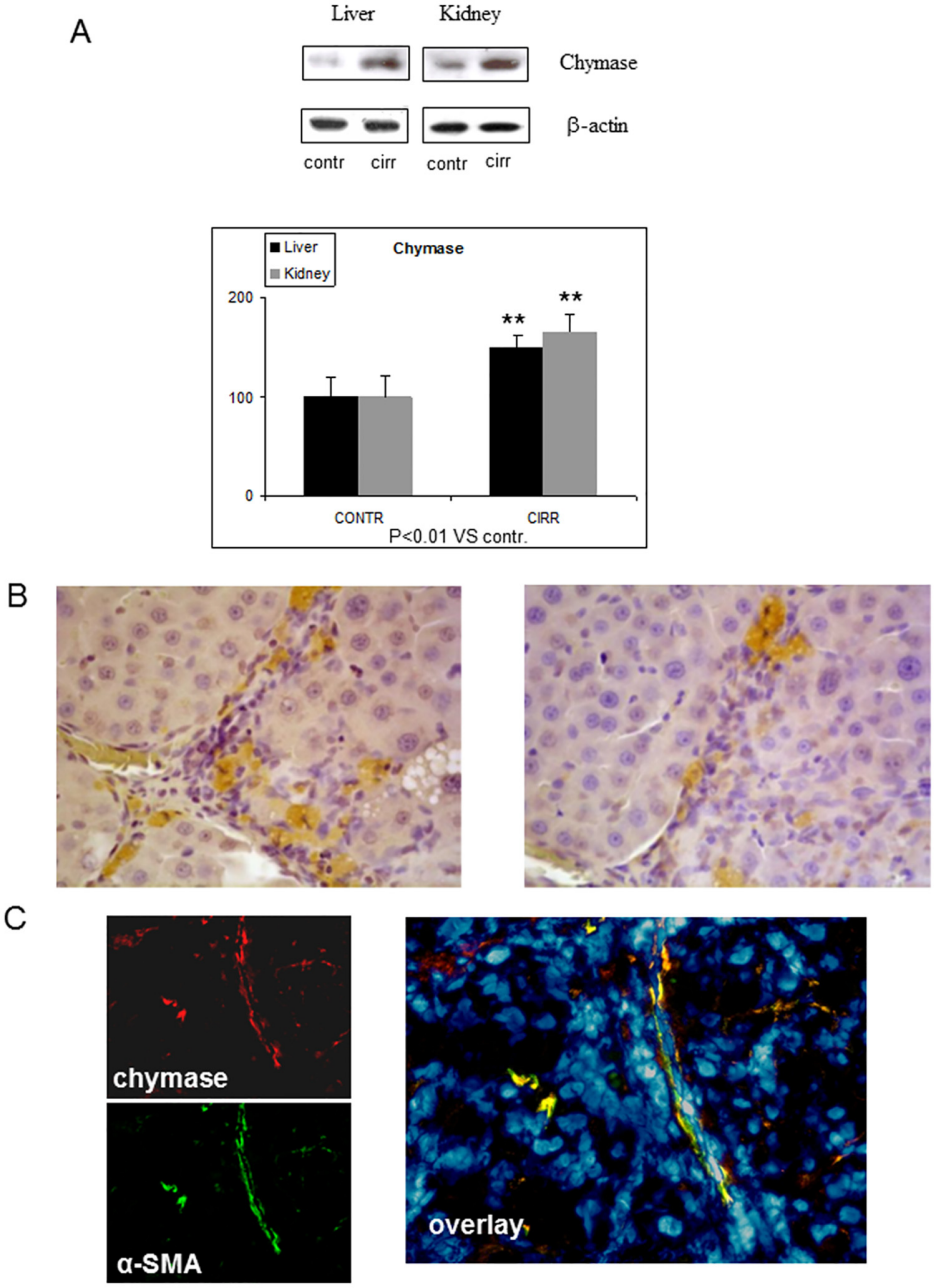
Panel A. Western blot of representative experiments showing chymase levels in tissue homogenate of liver and kidney of control (G1) and cirrhotic (G3) rats. β-actin is internal standard to evaluate non-specific protein expression in the homogenates. Panel B. Rat cirrhotic liver. Immunohistochemical location of chymase in close proximity to fibrotic septa and at the periphery of regenerative nodules (magnification: 40x). Panel C. Indirect immunofluorescence of hepatic distribution of chymase in rat cirrhotic liver. Chymase location in activated αSMA-positive myofibroblast-like cells in the fibrotic septa and at the periphery of regenerative nodules (magnification: 40x). Panel C includes: small images on the left side representing image acquisition of single fluorescence identifying chymase (red fluorescence) and αSMA (green fluorescence); a larger image (overlay, right side) offering electronic merging of fluorescent images plus correspondent nuclear staining (blue DAPI staining).

**Fig 6:**
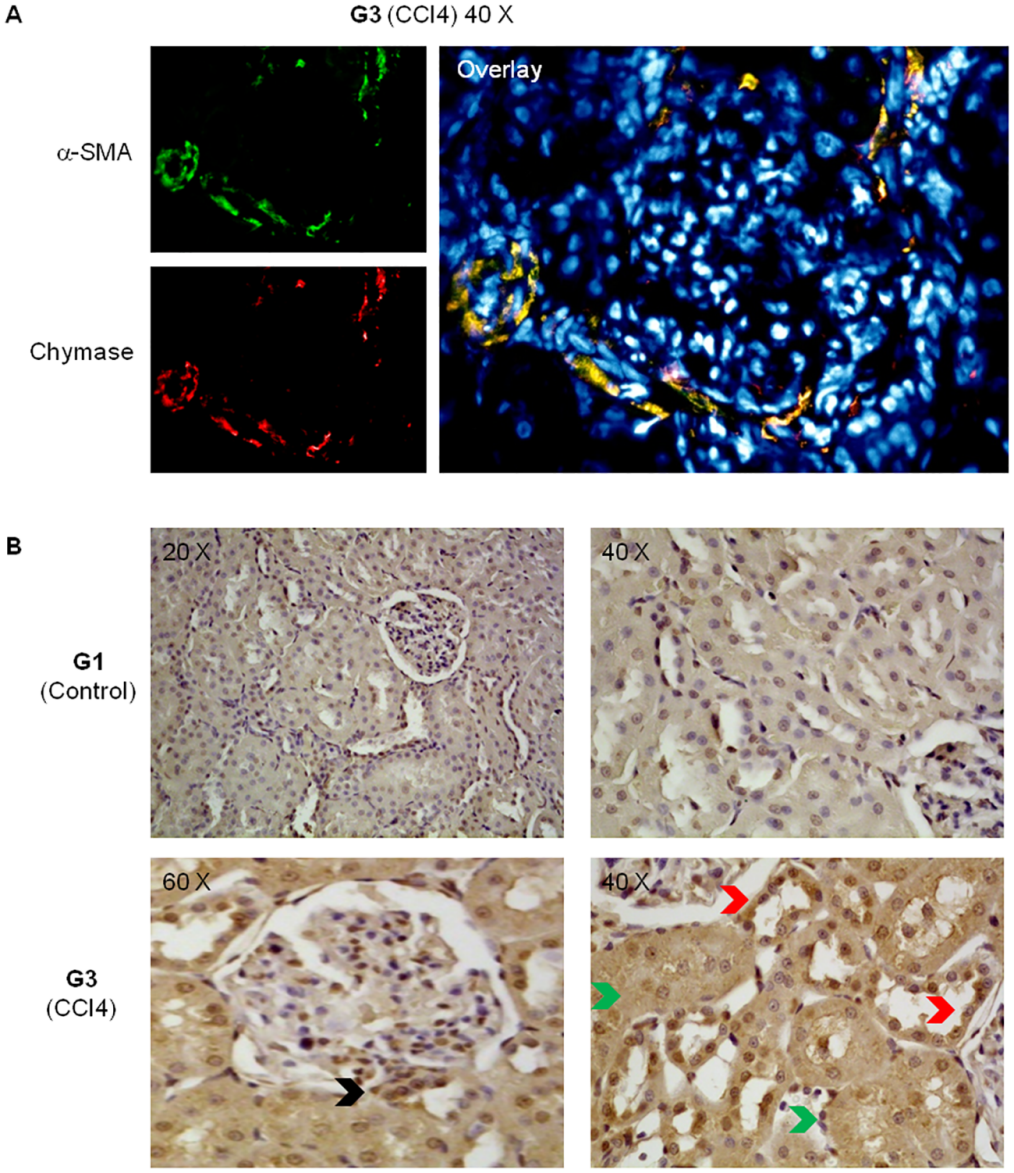
Renal distribution of chymase. Panel A: Indirect immunofluorescence staining of kidney sections from cirrhotic rats (G3 group). Panel B: Immunohistochemical staining for chymase in kidney sections from either control animals (G1) or cirrhotic rats (G3). In the kidney of cirrhotic rats, chymase was found in the wall of cortical arterioles (Panel A), in the wall of proximal convoluted tubules (Panel B, green arrows), in distal convoluted tubules (Panel B, red arrows), and at the vascular pole of the glomerulus (Panel B, black arrow).

**Fig 7:**
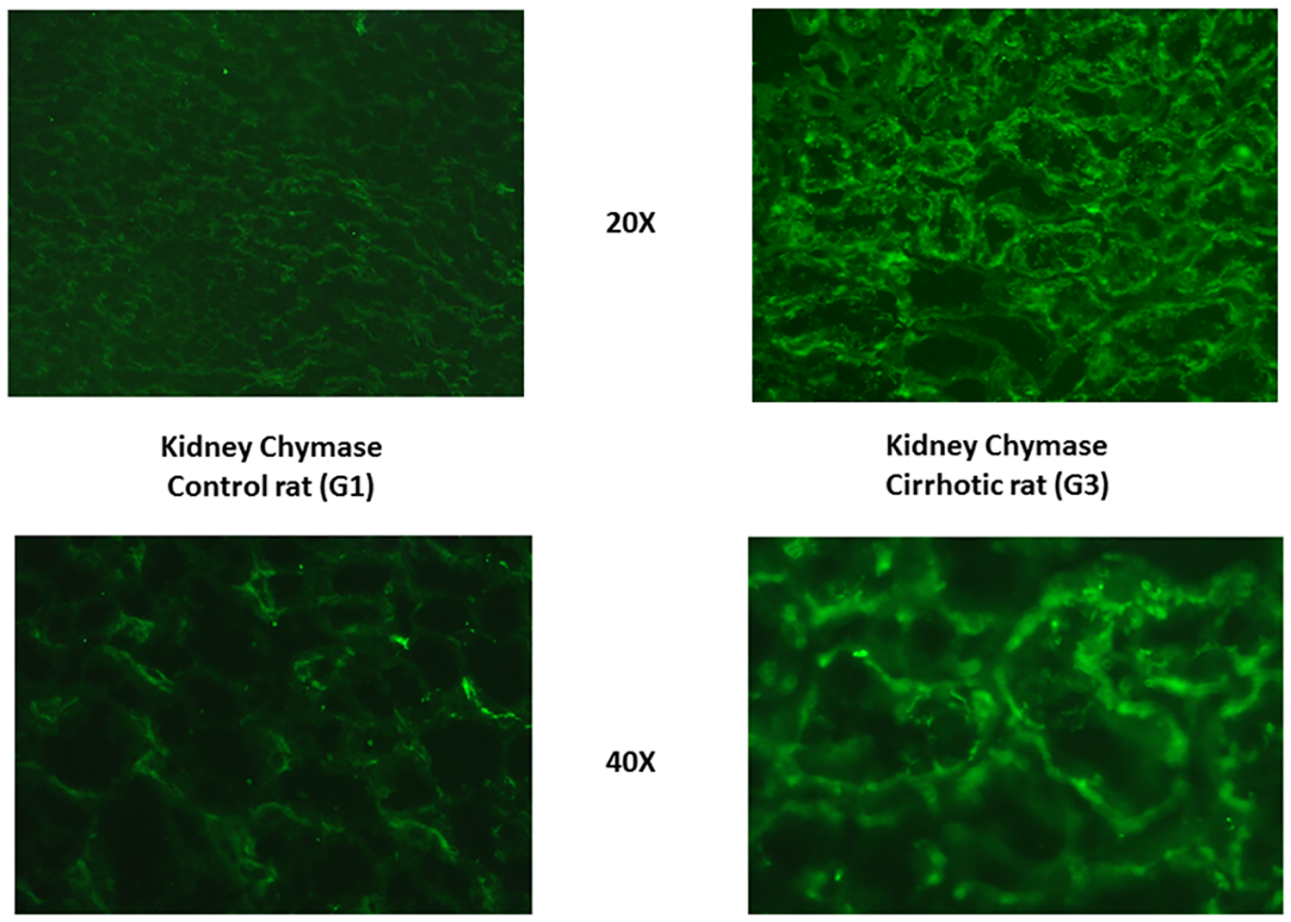
Renal chymase indirect immunofluorescence staining. Enhanced tubular expression of chymase in cirrhotic rats. Goat polyclonal anti-chymase primary antibodies (Santa Cruz Biotechnology, Inc.) and anti-goat C3y-conjugated secondary antibodies (Amersham Biosciences, Braunschweig, Germany) were used.

In liver and kidneys of G3 rats there was a larger amount of chymase than in normal rats. In the cirrhotic liver, chymase was found in activated αSMA-positive myofibroblast-like cells in the fibrotic septa and at the periphery of regenerative nodules (Fig 5). In the kidney of G3 rats chymase was located in the wall of the proximal and, to a larger extent, distal convoluted tubules, but also in cortical arterioles, identified through αSMA-positive staining, and at the vascular pole of the glomerulus (Figs 6 and 7).

### Liver and kidney function (Tables 1 and 2)

Portal pressure and serum bilirubin are lower and plasma albumin higher in G5 than in G3 rats. Inhibition of chymase reduced hepatic and renal levels of angiotensin II, endothelin-1 and TGF-β in G5 vs. G3 rats. Glomerular filtration rate, filtration fraction, urine flow rate, urinary sodium and potassium excretion rates, and fractional sodium excretion were increased in G5 rats compared to cirrhotic rats in G3. Tubular free-water reabsorption, a direct measurement of the tendence of the renal tubules to retain solute-free water, was reduced by chymase inhibition in G5 vs. G3 rats.

### Hormonal status and arterial pressure (Table 2)

G5 rats had normal levels of systemic plasma norepinephrine, vasopressin (ADH), Ang-II, and PRA, showing preserved effective arterial blood volume. Ascitic cirrhotic rats (G3) had significantly increased levels of these hormones in comparison with healthy controls. Arterial blood pressure was not significantly different in the five rat groups, with a tendency towards hypotension only in G3.

### Human liver chymase immunohistochemistry and *In vitro* cell expression (Figs 8 and 9)

**Fig 8:**
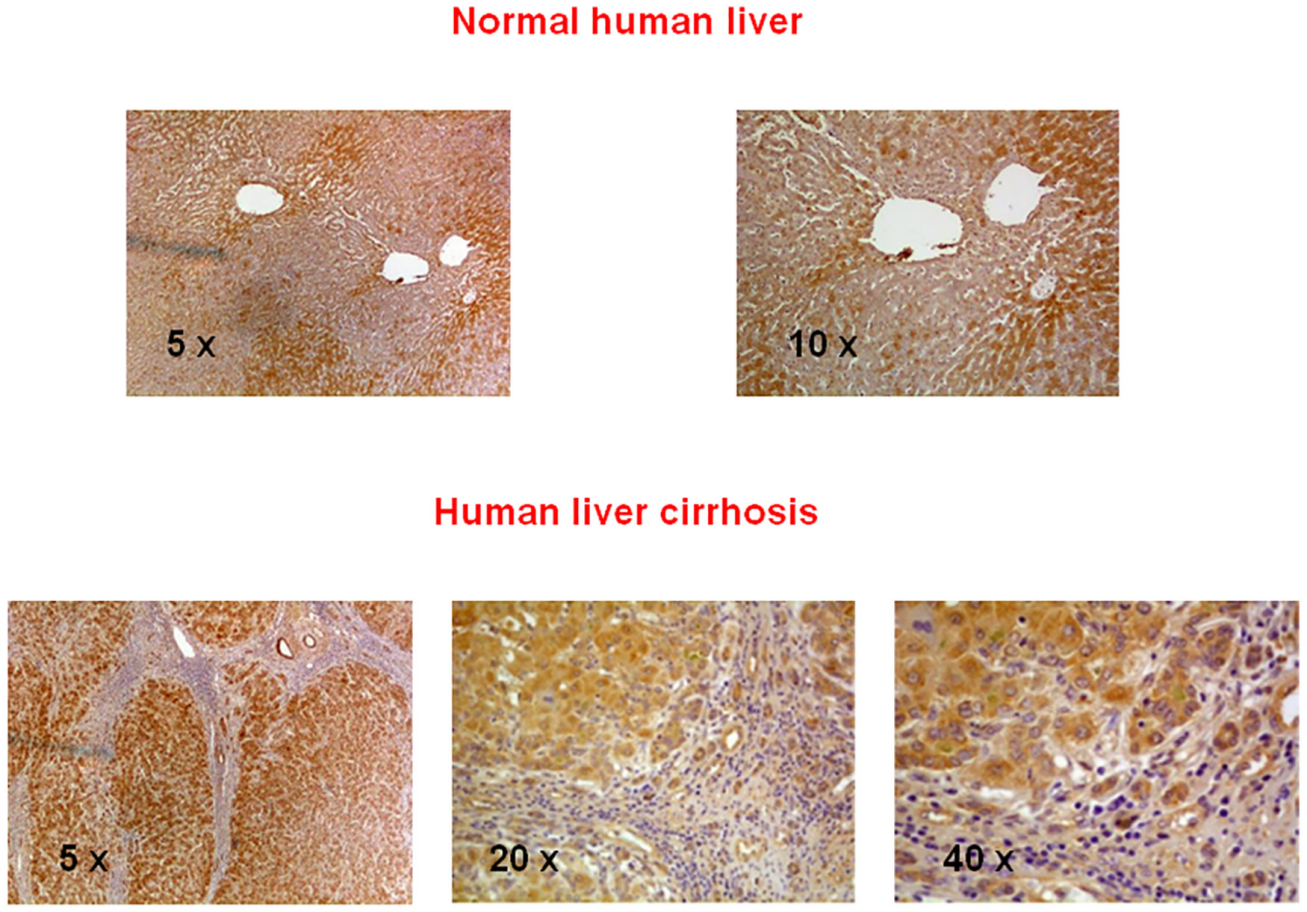
Chymase immunohistochemical localization in resected samples of normal and cirrhotic human liver. Original magnification as indicated.

**Fig 9:**
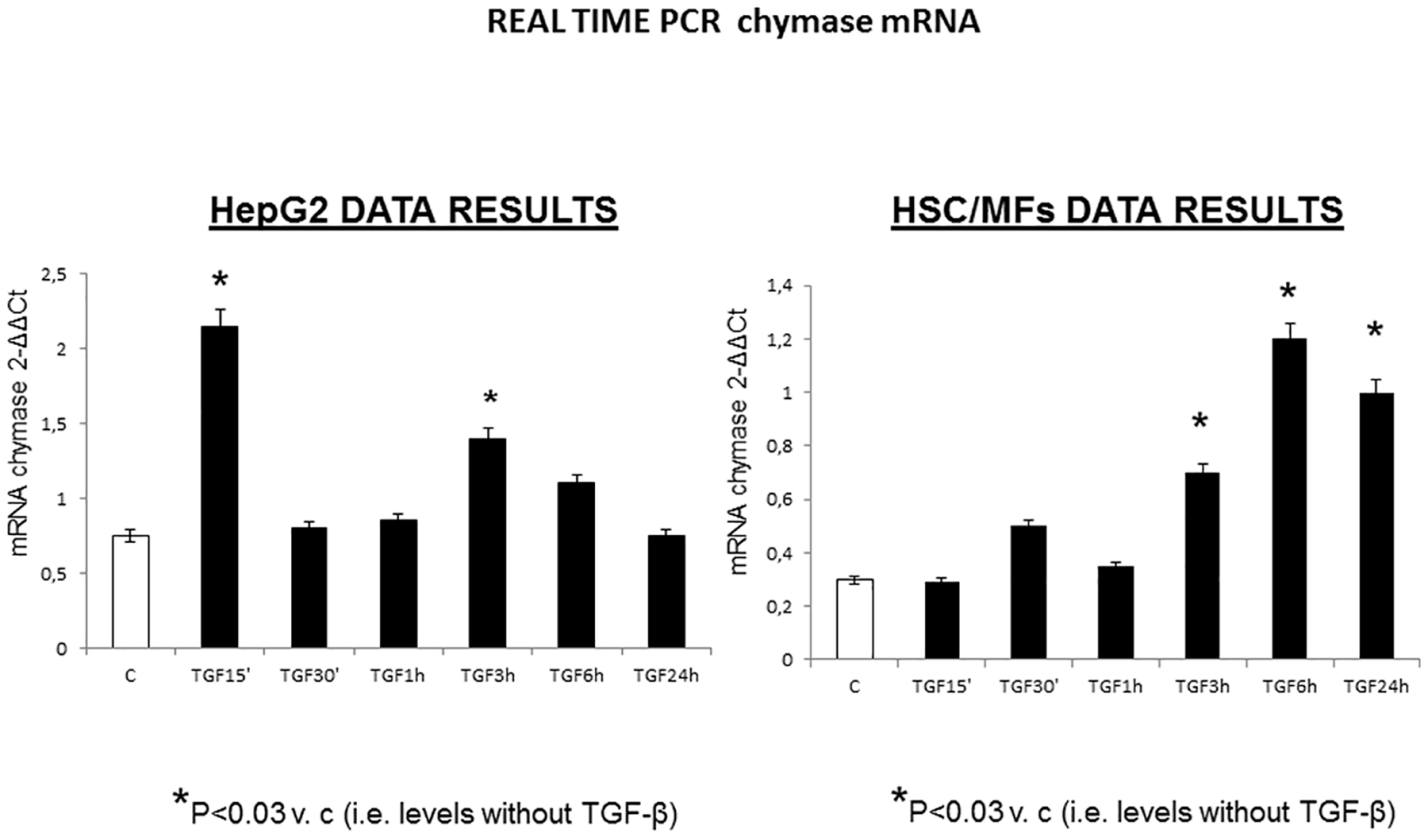
Both human activated, myofibroblast-like, hepatic stellate cells and human hepatoblastoma cells show basal expression of chymase mRNA and a considerable increase in its expression after stimulation with TGF-β1, earlier in HepG2 cells and later in activated stellate cells.

Normal human liver shows lobular metabolic zonation for chymase at the periphery of lobules. Conversely, hepatocytes of regenerative nodules contain a lot of chymase, especially near fibrotic septa. Myofibroblast-like cells at the periphery of larger septa contain chymase too, but to a lesser degree. Both human activated hepatic stellate cells and human hepatoblastoma cells show basal expression of chymase mRNA and a significant increase in its expression after treatment with TGF-β1.

## Discussion

Chymase inhibitor SF2809E, in this experimental setting, leads to prevention of histological cirrhosis and liver decompensation despite 13 weeks of CCl_4_ administration, which instead causes ascitic cirrhosis (Figs 1–4) with functional renal failure if chymase is not inhibited. Rats receiving SF2809E 20 mg/kg (G5) show lower portal pressure and hepatic levels of Ang-II, ET-1 and TGF-β, and improved liver function in comparison with the cirrhotic group receiving only CCl_4_ (G3) (Table 1). Moreover, in G5 rats, effective arterial blood volume is preserved and PRA, catecholamines, ADH and Ang-II levels are close to normal values (Table 2).

Chronic chymase inhibition reduces Ang-II, ET-1 and TGF-β levels also in the kidney, with ensuing improvement of GFR, filtration fraction, free-water clearance and natriuresis, in comparison with untreated cirrhotic rats (Table 2).

Key positions for Ang-II to influence liver matrix deposition and kidney function are those of chymase-positive cells: at the periphery of regenerative nodules in the cirrhotic liver (Fig 5) and in the wall of the renal proximal and distal tubules and in cortical arterioles (Figs 6 and 7). Moreover, hepatocytes of normal human liver host chymase at the periphery of lobules, but hepatocytes of regenerative nodules of cirrhosis contain an even larger amount of this enzyme (Fig 8) Accordingly, i*n vitro* human HepG2 cells and HSC/MFs do express chymase at baseline, with further transcription of chymase’s gene after profibrogenic TGF-β1 (Fig 9). This shows that in chronic liver diseases cytokines stimulate chymase overexpression, and chymase, in turn, may activate further pro-TGF-β to its active and fibrogenic form, as reported in literature [18]. Chymase up-regulation was reported also in human subjects with diabetic nephropathy [32] and in the ischemic kidney of two-kidney/one-clip hypertensive hamsters [33].

Up until now, in patients with chronic liver disease, a correlation between the number of chymase-containing mast cells and liver fibrogenesis was reported [34], and a decrease in the number of activated stellate cells was seen in chymase inhibitor-treated compared to placebo-treated hamsters receiving CCl_4_ [35].

Pharmacologic inhibition or genetic ablation of components of the systemic RAS attenuate experimental liver damage and fibrosis deposition by reducing the function of Ang-II [6, 36]. But ACE-inhibitors and AT1 receptor-antagonists aggravate the arterial hypotension and hyper-reninism of ascitic cirrhosis [37, 38].

Since 80% of angiotensin II-forming activity in kidney, heart and blood vessels is dependent on chymase [39], one might assume that chymase inhibitors, like ACE inhibitors, reduce arterial blood pressure and increase plasma renin. On the contrary, blood pressure is not lowered and renin not increased by chymase inhibitors in this and other studies [40]. This is due to ACE being located in endothelial cells and chymase in mast cells of the vascular adventitia. Moreover, systemic plasma contains effective serine protease (chymase) inhibitors [40].

Our group described, in experimental cirrhosis, the diuretic and portal hypotensive effects of the acute inhibition of metallo-endopeptidase neprilysin [22, 27], which degrades the natriuretic peptide angiotensin-(1–7) into angiotensin-(1–4) [41], destroys atrial natriuretic factors and generates endothelin-1 [27]. Now we are showing that hepatic αSMA-positive activated stellate cells, actual cell protagonists of liver fibrogenesis, express both neprylisin [22] and chymase, and therefore may both synthesize pro-fibrotic Ang-II and ET-1 and clear anti-fibrotic angiotensin-(1–7) and natriuretic peptides.

Finally, in chronic liver diseases, the concurrent overexpression of chymase in liver and kidney deserves further mention because production by this enzyme of fibrogenic vasoconstrictors and anti-natriuretic agents in both organs may well represent a further mechanism that makes hepatic and renal disorders so tightly linked in the natural history of liver cirrhosis.

## Abbreviations

ACE: angiotensin-converting enzyme
ADH: vasopressin
ALT: alanine aminotransferase
Ang-I: Angiotensin I
Ang-II: Angiotensin II
αSMA: α-smooth muscle actin
AST: aspartate aminotransferase
AT1: angiotensin type 1 receptor
CCl_4_: carbon tetrachloride
CIN: inulin clearance
CK: potassium clearance
CNa: sodium clearance
Cosm: osmolar clearance
CPAH: para-aminohippurate clearance
EIA: enzyme immunoassay
ET-1: endothelin-1
ET_A_: endothelin type A receptor
ET_B_: endothelin type B receptor
FEK: fractional excretion of potassium
FENa: fractional excretion of sodium
FF: filtration fraction
GFR: glomerular filtration rate
HCV: hepatitis C virus
HSC: hepatic stellate cell
IN: inulin
MAP: mean arterial pressure
MF: myofibroblast
N: norepinephrine
PAH: para-aminohippurate
Posm: plasma osmolality
PRA: plasma renin activity
RAS: renin-angiotensin system
RPF: renal plasma flow
TF-WR: tubular free-water reabsorption
TGF-β: transforming growth factor beta
Uosm: urinary osmolality
